# Fungal infections in burns: Diagnosis and management

**DOI:** 10.4103/0970-0358.70718

**Published:** 2010-09

**Authors:** Malini R. Capoor, Sujata Sarabahi, Vinay Kumar Tiwari, Ravi Prakash Narayanan

**Affiliations:** Department of Micrbiology Vardhman Mahaveer Medical College & Safdarjung Hospital, Delhi – 110 029, India; 1 Department of Burns & Plastic Surgery, Vardhman Mahaveer Medical College & Safdarjung Hospital, Delhi – 110 029, India

**Keywords:** Burns, fungal infections, moulds, yeasts

## Abstract

Burn wound infection (BWI) is a major public health problem and the most devastating form of trauma worldwide. Fungi cause BWI as part of monomicrobial or polymicrobial infection, fungaemia, rare aggressive soft tissue infection and as opportunistic infections. The risk factors for acquiring fungal infection in burns include age of burns, total burn size, body surface area (BSA) (30–60%), full thickness burns, inhalational injury, prolonged hospital stay, late surgical excision, open dressing, artificial dermis, central venous catheters, antibiotics, steroid treatment, long-term artificial ventilation, fungal wound colonisation (FWC), hyperglycaemic episodes and other immunosuppressive disorders. Most of the fungal infections are missed owing to lack of clinical awareness and similar presentation as bacterial infection coupled with paucity of mycology laboratories. Expedient diagnosis and treatment of these mycoses can be life-saving as the mortality is otherwise very high. Emergence of resistance in non-albicans Candida spp., unusual yeasts and moulds in fungal BWI, leaves very few fungi susceptible to antifungal drugs, leaving many patients susceptible. There is a need to speciate fungi as far as the topical and systemic antifungal is concerned. Deep tissue biopsy and other relevant samples are processed by standard mycological procedures using direct microscopy, culture and histopathological examination. Patients with FWC should be treated by aggressive surgical debridement and, in the case of fungal wound infection (FWI), in addition to surgical debridement, an intravenous antifungal drug, most commonly amphotericin B or caspofungin, is prescribed followed by de-escalating with voriconazole or itraconazole, or fluconazole depending upon the species or antifungal susceptibility, if available. The propensity for fungal infection increases, the longer the wound is present. Therefore, the development of products to close the wound more rapidly, improvement in topical antifungal therapy with mould activity and implementation of appropriate systemic antifungal therapy guided by antifungal susceptibility may improve the outcome for severely injured burn victims.

## INTRODUCTION

Burn wound infection (BWI) is a major public health problem and globally the most devastating form of trauma.[1] BWI is primarily caused by bacteria (70%) followed by fungi (20–25%), anaerobic and virus (5–10%). Fungi cause BWI as part of monomicrobial or polymicrobial infection, fungaemia, rare aggressive soft tissue infection and as opportunistic infections.[2]

Consequent to the availability of topical and systemic antimicrobial agents in 1960s, near eradication of universal BWI was witnessed. Subsequently, emergence of fungal infections in burn wound patients was seen.[1,2]

The omnipresence of fungi in environment coupled with suppression of normal bacterial flora promotes fungal superinfection in these patients. Though burn constitutes an important and independent risk factor for invasive fungal infection, most of the infections are misdiagnosed due to lack of clinical awareness and similar presentation as bacterial infection coupled with paucity of mycology laboratories. Nonetheless, early diagnosis and treatment of these mycoses can be life-saving as the mortality is otherwise very high.[3,4],[5] Emergence of resistance in non-albicans *Candida* spp., unusual yeasts and moulds in fungal BWI further complicates the scene.[6] Very recently, azole resistance in *Candida albicans* has been observed, leaving very few fungi susceptible to antifungal drugs, warranting antifungal susceptibility in such patients.[6,7]

In the developing countries, the scenario with regard to BWI is grim owing to lack of surveillance laboratories and dearth of well-equipped burn centres. Consequently, the clinical data pertaining to fungal BWI are scarce.[5,8]

There is worldwide decrease in bacterial infections due to better care of burn patients and availability of effective antibiotics. Consequently, the fungal BWI has shown an increasing trend.[9–14]

## EPIDEMIOLOGY

The incidence of fungal infection documented in literature is from 6.3 to 44% reported from various burn centres around the world.[3,9–11] However, a prior study from a largest burn centre in Asia done on 220 burn patients gave a 42% positivity rate for isolation of *Candida* spp. (colonisation) and a 10% as fungal wound infection (FWI).

Recently, there is change in the epidemiology of fungi despite the introduction of new antifungals.[15–19] Non-albicans *Candida* species have been found to be increasingly resistant against common antimycotic substances. Additionally, other species such as *Aspergillus* and Zygomycetes, with an aggressive and invasive infection are more frequently observed.[15,20,21]

Invasive and documented *Candida* infections have become a major cause of morbidity and mortality in burn patients with a prevalence of colonisation from 13 to 31.8%.[19] Immunosuppressive factors attributed include long-term use of antimicrobials (aminoglycosides, vancomycin, Central Venous Catheter (CVC)[2,5,14]

Moulds usually are seen as saprophytes in soil. In recent years, there is an increasing number of human infection due to *Aspergillus niger*[3,15,19,20] and *Fusarium* spp.,[15,22] mostly involving immunocompromised hosts causing localised infection, deep-seated skin infection and disseminated disease. Documents of *Fusarium* spp. infection in nonimmunocompromised hosts are infrequent and usually involve dialysis related or ocular infection.[21,22] *Fusarium* infection in burn wound is rare.[10,15,21] A recent history of nearby construction (<200 m) should be elicited in *Aspergillus* isolates of burn ward patients. Amongst Zygomycetes, only a single report documents isolation of Zygomycetes *Syncephalesrtum* from burn wound patient. Nonetheless, there are reports of *Mucor* spp. isolation from extensive burn wounds. Human zygomycosis caused by mucorales occur as opportunistic infections. Host risk factors include diabetes mellitus, neutropenia, sustained immunosuppressive therapy, steroids, iron chelation therapy, broad-spectrum antibiotic use, severe malnutrition, burns and wounds. Due to development of burn stress pseudodiabetes (persistent hyperglycaemia and glucosuria), the chances of acquiring Zygomycetes infection is high.[23–25]

## AETIOPATHOGENESIS AND RISK FACTORS

Fungal infections usually occur after the second week of thermal injury, usually following a period of burn wound colonisation with fungi found in the surrounding environment and/or in the case of *Candida* spp. from the patients own flora.[1,5],[26]

In burn patients with fungal infection, mortality is associated with the presence of fungaemia, multiple positive cultures and invasion of healthy skin.

The risk factors for fungal infection in burns are age of patient, total burn size, BSA (30–60%), full thickness burns, inhalational injury, prolonged hospital stay, late surgical excision, open dressing, artificial dermis, central venous catheters, antibiotics (imipenem, vancomycin, aminoglycosides), steroid treatment, long-term artificial ventilation, fungal wound colonisation (FWC), hyperglycaemic episodes and other immunosuppressive disorders.[1,2,11,15–18] In 3–21% of burn patients, inhalational injury occurs and is usually proportional to depth and total body surface area (TBSA) burned.[1,2] Inhalation of smoke causes toxic and chemical injury to tracheobronchial epithelium, causing ventilation perfusion mismatch and acute respiratory distress syndrome, thereby increasing the mortality. In a previous study from American Burn Association, researchers reported a fungal positivity of 6.3%, and 38% patients had inhalational injuries[11] Similarly, in a prior study, the figures were 6.4 and 14%, respectively.[2] In another study, Hovarth *et al*.[2] concluded that fungal infection has more than 3 times the impact of inhalational injury on mortality, and TBSA (30–60%), full thickness burn size (FTBS) positivity, age (curvilinearly) and inhalational injury (positivity) contributed independently to the probability of death. Prior studies have reported a mortality ranging from 21.2% to 76%.[2,11,14,27]

None of the prior trials have recommended antifungal prophylaxis in burn patients due to the possible development of resistances and increasing costs.[9,11,18] It is well documented that burn wounds complicated by fungal infections constitute an independent predictor for mortality in patients with a burned TBSA of 30–60%. On the either side of 30–60% range of TBSA, contributions of other variables are taken into consideration.[2,9] However, a prior study observed that the average TSBA for fungal infections in burns was 28%.[26] A prior study observed that patients with *Candida* spp. infection had received imipenem, vancomycin or an aminoglycoside.[14]

## CLINICAL FEATURES

The diagnostic methods to identify mycoses are conventional and often specific to some organisms. Direct tissue biopsy is performed rarely and mostly in case of a justified suspicion. The growth of fungal cultures is unreliable and associated with considerable latency – sometimes too late for the clinician to initiate antimycotic therapy appropriately.[15]

Since burn patients usually present with symptoms of Systemic Inflammatory Response Syndrome (SIRS), clinical warning signals masquerade as bacterial infection. There is need for a re-evaluation of definitions of SIRS and sepsis, as previously published.[15]

At the outset, to suspect a fungal infection, on admission, burn sizes should be estimated directly as a percentage of the BSA in a standard fashion using Lund-Browder charts. Patients present with local signs and symptoms of FWI like separation of eschar, partial thickness burn converting into full thickness burn, blackening of tissue, worsening of wound with cellulitis or necrotising fasciitis. Clinically, patients with fever, despite the intake of broad-spectrum antibiotics for >7–15 days, and deteriorating condition in the presence of risk factors should be suspected of fungal infection.

## LABORATORY DIAGNOSIS

Time interval of collecting burn wound sample: Full thickness burns should be excised at 7th, 14th, 21st days and ≥28 day.

Specimens for fungal culture in burns: for demonstration of FWI, tissue biopsy (living tissue including dermis) is done from under the eschar (0.5 g wt.) multiple sites (at least three sites) and multiple times (at least three times). Culture of the tissue biopsy is interpreted as per Buchanen *et al*.,[28–30] using semiquantitative method [>10^5^ colony forming units (CFU)/g tissue] by using the formula:

$$\frac{\mathrm{CFU}\;\times \;\log\;\mathrm{reciprocal}\;\times \;2}{\mathrm{tissue}\;\mathrm{wt}.\mathrm{(g)}}$$

Relevant samples like fine needle aspirate of subeschar exudate should be collected, and pus swab from surgical site, blood culture for fungus, urine, throat swabs are collected to look for colonisation.

Deep tissue biopsy and other relevant samples are processed by standard microbiological procedures using direct microscopy (Gram’s Stain, KOH) and culture.[1,2,27] Specimens in which fungal elements are observed on KOH mount or Gram’s stain are further confirmed by histopathological examination by using periodic acid Schiff ’s stain.

Samples are inoculated on various mycological media (Sabouraud’s dextrose agar with and without choloramphenicol) in duplicate tubes and should be incubated at 25 and 37°C for up to 6 weeks before giving a negative culture report. Blood agar and Mc Conkey’s agar are incubated at 37°C for 24 hours to rule out bacterial infection.

Identification of yeasts is carried out using germ tube test, characteristics on corn meal agar, cultural characteristics on Hichrome agar (Himedia, Mumbai, India), tetrazolium reduction test, carbon and nitrogen assimilation test and by using API 20 C yeast identification strip (Biomeriux, Marcy, France). Moulds are identified using lactophenol cotton blue mount preparation for conidiogenesis, pattern and arrangement. Identification of nonsporulating mould is carried out using slide culture with potato dextrose agar.

### Specimen classification

Each patient’s fungal status category is defined according to the deepest level. FWI is defined as invasion of fungal elements into viable tissue. FWC is defined as fungal elements in eschar (non-viable burnt skin) or neo-eschar (previously excised and now necrotic wound surface but not viable tissue).

### Sterile specimen

No fungal growth or absence of fungal elements.

The antifungal susceptibility of the yeasts is tested by E-strip or broth micro-dilution against amphotericin B, fluconazole, itraconazole, voriconazole and caspofungin as per CLSI recommendation.[30] For mould infection, the antifungal susceptibly was tested by E-strip against amphotericin B.

## SYSTEMIC AND LOCAL MANAGEMENT

It is crucial to speciate fungi as regards the management with topical and systemic antifungal therapy. Patients with FWC should be treated by aggressive surgical debridement and, in the case of FWI, in addition to surgical debridement, an intravenous antifungal drug, most commonly amphotericin B or caspofungin, is prescribed followed by de-escalation with voriconazole or itraconazole, or fluconazole depending upon the species or antifungal susceptibility, if available.

Isolation of a non-albicans *Candida* spp. calls for amphotericin B or voriconazole or caspofungin therapy. If the patient is renal compromised, liposomal amphotericin or caspofungin is recommended. Among the newer available antimycotic substances, echinocandins and triazoles show advantages compared to conventional imidazol-based azoles and polyenes concerning efficacy, specificity, safety and patient compliance.[2,30],[31] Promising results are to be expected by *Candida* secretory aspartic protease (SAPs) inhibitors and calcineurin signalling pathway blockers.[2]

*C. albicans* is usually susceptible to azoles. Recently, there are reports of emergence of azole resistance in *C. albicans* from Indian subcontinent.[6,7] For an *Aspergillus* isolate, use of amphotericin B (alone or in combination of voriconazole) or caspofungin is recommended. Higher cost precludes the routine use of liposomal amphotericin B or caspofungin. For a Zygomycetes or Fusarial infection, amphotericin B is recommended.[1,18]

### Local treatment

Local treatment with nystatin, a topical antifungal, is needed in a lower concentration (3 *μ*g/ml) if *C. albicans* is isolated, and a higher concentration (6.25 *μ*g/ml) is needed to inhibit other *Candida* spp. All the more, angioinvasive fungal infections like those due to *Aspergillus* and *Fusarium* spp. require >6 million units/g.[1,3,12] A large number of studies have proven that large-scale use of mafenide acetate[32] favours the overgrowth of fungi.[1,33] Therefore, a combination of mafenide and nystatin can be used to prevent superinfection.[1,3,12] Nonetheless, mafenide is not used in some centres. However, a prior study evaluated the antimicrobial activity of ACTICOAT Antimicrobial Barrier Dressing, a silver-coated wound dressing, and compared it with silver nitrate, silver sulphadiazine, and mafenide acetate showed that the former dressing killed the bacteria much faster. The study also suggested that a single susceptibility test such as a minimum inhibitory concentration (MIC) or zone of inhibition test does not provide a comprehensive profile of antimicrobial activity of a topical antimicrobial agent or dressing. Therefore, a combination of tests is desirable.[33]

### Surgical management

Immediate and extensive wound debridement and early coverage of wound defect, preferably with allografts, are the surgical approach to the treatment. The coverage of a burn area by skin grafts or flaps should be performed only when wound infection is controlled (negative swab cultures and biopsies) and there is no clinical evidence of fungal sepsis.[1–3] However, despite the introduction of new antimycotic substances, some fungal organisms preserving angioinvasive and proteolytic potential still require radical surgical therapy to provide a chance for survival. The restoration of immune function, early surgical intervention and early wound closure gain a key function in limiting the risk of fungal infection in burn patients.[2,15,16]

### Role of hospital infection control in fungal burn wound infection

The data on role of hospital infection control in fungal wound infection are scarce.[5,8,12,17,18] There are limited studies showing corroboration of hospital environmental strains of fungi versus strains isolated from patients.[5,8] A study from National Mycology Referral Laboratory at PGIMS, Chandigarh, was done on 25 severely burnt patients and their surroundings. The environmental sampling revealed fungal contamination by dematiaceous hyphomycetes, *Aspergillius, Penicillium, Fusarium* and *Candida* spp., whereas the colonising or invading fungi from the patients were *Aspergillus flavus* and *Candida* spp. This study highlighted the pathogenic potential of some of the environmental fungal isolates located in the vicinity of the immunocompromised patients and stresses the need for decontamination of the environment of the burn care unit.

In a molecular study,[5] a total of 228 different *Candida* species were obtained from various body locations of burn patients. Species identification revealed that *C. albicans* was the most predominant followed by *Candida tropicalis, Candida glabrata, Candida parapsilosis, Candida krusei* and *Candida kefyr*. DNA fingerprinting of all *C. albicans* isolates was done by using *CARE-2* probe. Fingerprinting analyses of all the *C. albicans* strains revealed that strains collected from different patients were different. It was concluded that patients with disseminated candidiasis had a similar, but unique strain isolated from all body locations. This study depicted that commensal isolates might be turning pathogenic. Nonetheless, more studies are needed to support or refute this claim.

With the advent of newer antifungal therapies with less toxicity than the conventional ones like amphotericin B, the importance of accurate prediction models of fungal burn wounds is paramount.

The propensity for fungal infection increases, the longer the wound is present. Therefore, the development of products to close the wound more rapidly, improvement in topical antifungal therapy with mould activity and implementation of appropriate systemic antifungal therapy guided by antifungal susceptibility improves the outcome for severely injured burn victims susceptible to fungal infection. All the more, infection control practices should be followed and microbiological surveillance data should be used frequently to monitor the trend, as the treatment is expensive.

## References

[1] Church D, Elsayed S, Reid O, Winston B, Lindsay R (2006). Burn wound infections. Clin Microbiol Rev.

[2] Horvath EE, Murray CK, Vaghan GM, Chung KK, Hospenthal DR, Wade CE (2007). Fungal wound infection (not colonization) is independently associated with mortality in burn patients. Ann Surg.

[3] Mousa HA (1999). Fungal infection of burn wound in patients with open and occlusive treatment methods. East Mediterr Health J.

[4] Tsoutsos D, Tsati E, Metaxotos N, Keramidas E, Rodopoulou S, Ioannovich J (2001). Extensive burn injury complicated by mucormycosis: A case report. Ann Burns Fire Disasters.

[5] Gupta N, Hague A, Lattif AA, Narayan RP, Mukhopadhyay G, Prasad R (2005). Epidemiology and molecular typing of Candida isolates from burn patients. Mycopathologia.

[6] Chakrabarti A, Chatterjee SS, Rao KL, Zameer MM, Shivaprakash MR, Singhi S (2009). Recent experience with fungemia: Change in species distribution and azole resistance. Scand J Infect Dis.

[7] Pfaller MA, Diekema DJ, Messer SA, Boyken L, Hollis RJ (2003). Activities of fluconazole and voriconazole against 1,586 recent clinical isolates of Candida species determined by broth microdilution, disk diffusion and E-test method: Report form the ARTEMIS Global Antifungal Susceptibility Program, 2001. J Clin Microbiol.

[8] Chakrabarti A, Nayak N, Kumar PS, Talwar P, Panigarhi D, Chari PS (1992). Surveillance of nosocomial fungal infections in a burn care unit. Infection.

[9] Murray C, Loo F, Hospenthal D, Cancio L, Jones J, Kim S (2008). Incidence of fungal infections and related mortality following severe burns. Burns.

[10] Becker WK, Cioffi WG, McManus AT, Kim SH, McManus WF, Mason AD (1991). Fungal burn wound infection: A 10 year experience. Arch Surg.

[11] Ballard J, Edelman L, Saffle J, Sheridan R, Kagan R, Bracco D (2008). Multicentre Trials Group. American Burn Association. J Burn Care Res.

[12] Mousa HA (1997). Aerobic anaerobic and fungal burn wound infections. J Hosp Infect.

[13] Schofield CM, Murray CK, Horvath EE, Cancio LC, Kim SH, Wolf SE (2007). Correlation of culture with histopathology in fungal burn wound colonization and infection. Burns.

[14] Cochran A (2002). Systemic *Candida* infection in burn patients: A casecontrol study of management patterns and outcomes. Surg Infect (Larchmt).

[15] Greenhalgh DG, Saffle JR, Holmes JH, Gamelli RL, Palmieri TL, Horton JW (2007). American Burns Association Consensus Conference to define sepsis and infection in burns. J Burn Care Res.

[16] Mathew BP, Nath M (2009). Recent approaches to antifungal therapy for invasive mycoses. ChemMedChem.

[17] Dries DJ (2009). Management of burn injuries - recent developments in resuscitation, infection control and outcomes research. Scand J Trauma Resusc Emerg Med.

[18] Struck MF (2009). Infection control in burn patients: Are fungal infections underestimated?. Scand J Trauma Resusc Emerg Med.

[19] Macedo JL, Santos JB (2005). Bacterial and fungal colonization of burn wounds. Braz J Infect Dis.

[20] Stone HH (1979). Aspergillus infection of the burn wound. J Trauma.

[21] Burdge JJ, Rea F, Ayers L (1988). Non-candidal fungal infections of burn wound. J Burn Care Rehabil.

[22] Paz RN, Strahilevitz J, Shapiro M, Keller N, Goldshmied-Reoven A, Yarden O (2004). Clinical and epidemiological aspect of infectious caused by Fusarium spp.: A collaborative study from Israel. J Clin Microbiol.

[23] Ribes JA, Vanover-Sams CL, Baker DJ (2000). Zygomycetes in human disease. Clin Microbiol Rev.

[24] Tang D, Wang W (1998). Successful cure of an extensive burn injury complicated with mucor wound sepsis. Burns.

[25] Constantinides J, Misra A, Nassab R, Wilson Y (2008). *Absidia corymbifera* fungal infection in burns: A case report and review of literature. J Burn Care Res.

[26] Caetano M, Ramos S, Abreu J, Casalta J, Pinheiro S, Diogo C Fungal infections at a Coimbra burns unit: 2003–2007 Abstract number: R2459. 18th European Congress of Clinical Microbiology and Infectious Diseases Barcelona, Spain, 19–22 April 2008.

[27] Forbes BA, Sahm DF, Weissfeld L, Eissfeld AS (2002). Bailey and Scott.s Diagnostic Microbiology.

[28] Buchanan K (1986). Comparison of quantitative and semiquantitative culture techniques for burn biopsy. J Clin Microbiol.

[29] Liu MZ (1992). Need for more emphaisis on fungal infections in burn patients. Zhonghua Wai ke Za Zhi.

[30] (2002). National Committee for Clinical Laboratory Standards 2002. Reference method for broth dilution testing of yeast approved standard. M27-A2.

[31] Pappas PG, Kauffman CA, Andes D, Benjamin DK, Calandra TF, Edwards JE (2009). Clinical practice guidelines for the management of Candidiasis: 2009 Update by the Infectious Disease Society of America. Clin Infect Dis.

[32] Patrice W (2008). Mafenide tied to fungal infections in burn patients (Wound Healing). Skin Allergy News.

[33] Yin HQ, Langford R, Burrell RE (1999). Comparative evaluation of the antimicrobial activity of acticoat antimicrobial barrier dressing. J Burn Care Rehabil.

